# Feedback learning opportunities from medical student logs of paediatric patients

**DOI:** 10.1186/s12909-019-1533-y

**Published:** 2019-04-11

**Authors:** Helen M. Wright, Moira A. L. Maley, Denese E. Playford, Pam Nicol, Sharon F. Evans

**Affiliations:** 1 The Rural Clinical School of Western Australia, Faculty of Health and Medical Sciences, University of Western Australia, M706, 35 Stirling Highway, Crawley, WA 6009 Australia; 2 Division of Paediatrics, Faculty of Health and Medical Sciences, University of Western Australia, M501, 35 Stirling Highway, Crawley, WA 6009 Australia; 3 Department of General Paediatrics, Perth Children’s Hospital, 15 Hospital Ave, Nedlands, WA 6009 Australia

**Keywords:** Feedback, Logs, Paediatric, Rural

## Abstract

**Background:**

Feedback can alter medical student logging practices, although most learners feel feedback is inadequate. A varied case mix in rural and urban contexts offers diverse clinical encounters. Logs are an indicator of these clinical experiences, and contain opportunities for feedback, which can greatly influence learning: we labelled these ‘feedback learning opportunities’ (FLOs).

We asked:

- How often do FLOs occur?
- What are the case complexities of rural compared to urban paediatric logs?
- Do more complex cases result in more FLOs?

**Methods:**

In Western Australia, 25% of medical students are dispersed in a Rural Clinical School (RCSWA) up to 2175 miles (3500 km) from the city. Urban students logged 20 written cases; rural students logged a minimum of 25 paediatric cases electronically. These were reviewed to identify FLOs, using a coding convention. FLO categories provided a structure for feedback: medical, professionalism, insufficient, clinical reasoning, student wellbeing, quality and safety, and sociocultural. Each log was assigned an overall primary, secondary or tertiary case complexity.

**Results:**

There were 76 consenting students in each urban and rural group, providing 3034 logs for analysis after exclusions. FLOs occurred in more than half the logs, with significantly more rural (OR 1.35 95% CI 1.17, 1.56; *p* < 0.0001). Major FLOs occurred in over a third of logs, but with no significant difference between rural and urban (OR 1.10 95% CI 0.94, 1.28; *p* = 0.24). Medical FLOs were the most common, accounting for 64.0% of rural and 75.2% of urban FLOs (OR 1.71 95% CI 1.37, 2.12; *p* < 0.0001). Students logged cases with a variety of complexities. Most cases logged by urban students in a tertiary healthcare setting were of primary and secondary complexity. Major medical FLOs increased with increasing patient complexity, occurring in 32.1% of tertiary complexity cases logged by urban students (*p* < 0.001).

**Conclusions:**

Case logs are a valuable resource for medical educators to enhance students’ learning by providing meaningful feedback. FLOs occurred often, particularly in paediatric cases with multiple medical problems. This study strengthens recommendations for regular review and timely feedback on student logs. We recommend the FLOs categories as a framework for medical educators to identify FLOs.

## Background

Feedback on cases that students have encountered is central to, and can greatly influence, learning. Feedback has been defined as information provided about some aspect of the appraisee’s performance or understanding [1]. Feedback has been shown to alter medical student logging practices [2], although most learners don’t feel they get adequate or effective feedback [3].

We have previously reported alignment of the quantity and variety of paediatric presentations logged by rural and urban medical students with Australian paediatric hospital admissions [4]. Review of student logs during the earlier research highlighted that case logs contain numerous opportunities for medical educators to provide valuable feedback to students. We chose to name these instances ‘feedback learning opportunities’ (FLOs) for this study. We believe such opportunities constitute a valuable addition to case-based teaching, but the extent to which these FLOs occur is unknown. The present paper provides detail on FLO frequency and severity.

In Australia, as in other countries, medical students are taught in rural and urban settings [5]. These settings provide medical students with exposure to a varied case mix, including different case complexities, essential to support clinical learning [6, 7]. We wanted to know whether the diverse settings and case complexities reflected differences in FLOs.

Understanding the degree to which FLOs were present in urban vs rural case logs, and how this relates to case complexity, could help guide faculty development for the recognition of FLOs and their use in student education. We therefore designed this study to provide an evidence base about the potential educational value of logs as a resource for medical educators to provide feedback to students.

We asked:

- How often do feedback learning opportunities (FLOs) occur in medical student logs?
- What are the case complexities of rural compared to urban paediatric logs?
- Do more complex cases result in more feedback learning opportunities (FLOs)?

The results would primarily inform practice in the Australian context, however, as other countries have both rural and urban settings, the findings will have implications for other disciplines, institutions and clinical contexts.

## Methods

### Study design

This study is a retrospective audit of paediatric logs entered by all consenting medical students from an established medical school at the University of Western Australia (UWA). Consent, ethics approval, inclusions, exclusions and log categorisation were detailed in the previous paper [4].

### Participants and settings

In Western Australia, 25% of medical students are widely dispersed up to 2175 miles (3500 km) from the university’s city campus in a Rural Clinical School (RCSWA), for one academic year. RCSWA sites are all classified as rural or remote [8], with students predominantly community based.

### Logging instructions to students

Urban students were required to log and submit 20 paediatric cases in a written logbook. They were instructed to note clinical problems, history, examination, investigations, working diagnosis and differentials, a problem based management plan and reflection on learning. Specific requirements for cases logged were two outpatients; one Aboriginal child, where it was essential to apply holistic case management; and two developmental assessments.

Rural students were required to log a minimum of 25 paediatric cases electronically using a custom-built web database. Cases were from the core paediatric presentations in the guidebook. Specific requirements for cases logged were 10% Aboriginal cases; and 10% to include personal and professional development topics. Details included patient demographics, where students encountered cases, the clinical problem(s), differential diagnosis and formulation and follow-up. Case logs were to be discussed monthly with a medical educator, with three timetabled reviews during the year to demonstrate adequate numbers and quality of cases.

Satisfactory completion of these logged cases was a barrier assessment for urban and rural students.

### Outcomes measured

To answer the study questions we measured:

- Frequency, categories and severity of FLOs in logs of paediatric cases.
- Primary, secondary and tertiary levels of complexity of cases logged.

### The coding convention

As an outcome of iterative reading of multiple logs, described in an earlier paper, a coding convention was created by the main author (HW), who reviewed all case logs. The convention is described below, and includes criteria for identifying and categorising different kinds of FLOs.

### Definition of feedback learning opportunities (FLOs)

Logged cases were reviewed by HW, using the coder’s experience as a medical educator, to identify where feedback could be provided to students. Since medical students are known to under-recognise diagnoses [9], they may need specific feedback from teachers to confirm appropriate entries, advise if more information is required, consider all clinical problems for each patient, develop clinical reasoning skills [7], correct inaccuracies [10], restructure understandings, or provide alternatives to learning [1]. Accordingly, FLO categories were developed to assist medical educators with a structure for feedback when reviewing student logs. These were: medical, professionalism, insufficient, clinical reasoning, student wellbeing, quality and safety, and sociocultural.

### Case complexity

Each log was assigned an overall primary, secondary or tertiary case complexity for the whole case by HW. Coding conventions for complexity were that if a logged case needed subspecialist input – eg. diabetes, paediatric surgery, tertiary visiting outpatients then it was coded as tertiary complexity. If the case could have been admitted to a secondary hospital under care of the paediatrics team, or could have been seen in a general paediatric outpatient clinic then it was coded as secondary complexity. If the case could be managed by a GP then it was coded as primary complexity. Newborns were coded as primary complexity if uncomplicated; secondary if involvement of the paediatric team was required; tertiary if neonatal care or transfer was required.

### Procedures

#### Criteria for each FLO category

FLO categories developed by HW were labelled as Medical FLOs: where the student should have considered all clinical problems of the case but didn’t; as Professionalism FLOs: where logs suggested the student may have benefited from a discussion about professional issues or behaviours with a medical educator; as Insufficient FLOs: where more information was required to interpret the case; as Clinical reasoning FLOs: where there was no working diagnosis, or incomplete differential diagnosis; as Student wellbeing FLOs: where the log raised concerns about student welfare; as Sociocultural FLOs: where students needed to log relevant social history; as Quality and safety FLOs: where cases had a potential adverse outcome, or where inaccuracies should be corrected. Where appropriate, more than one FLO was allocated, for example clinical reasoning and quality and safety where a missing differential diagnosis had the potential for a poor patient outcome.

#### FLO severity

Appropriate logs where feedback was not required were coded as no FLO. We used ‘could’ or ‘should’ as terms to determine FLO severity, defined as minor or major. FLOs were determined as major if a student should have had recommendations made by a teacher about the content of the case logged, as the FLO was sufficient to warrant feedback. Minor FLOs included simple omissions or lack of information, where a medical educator could have provided some feedback, however this was desirable not a necessity. For example, an infant with a first ear infection where a student could have assessed development would have a minor medical category allocated; an infant with recurrent ear infections and glue ear where a student should have assessed development to look for speech and language delay would have had a major medical category allocated. Further examples and scoring are provided in Appendix 1.

### Statistical analysis

Excel data were imported into SAS (SAS Institute Inc. Cary, NC, USA V9.4). Rural-urban comparisons were made using Chi square and Fisher’s exact test for frequency data. The cell X^2^ test was used for clarification to identify parts of tables where dependencies between row and column categories may exist.

Inter- and intra-rater agreement for patient complexity and major FLOs between HW (previously a rural paediatrician, currently an urban specialist general paediatrician) and an experienced rural general paediatrician was undertaken on a subset of 1% of case logs.

Urban/rural inter-rater agreement for complexity was measured using a weighted Cohen’s Kappa coefficient, as complexity was ordered from primary to secondary to tertiary. Agreement for whether a student had a major FLO was measured using a simple Kappa coefficient.

## Results

Seventy six students gavewritten consent in each of the urban and rural groups (76/107 urban and 76/77 rural). After exclusions (cases seen in rural general practices, adults, antenatal cases and duplicates) there were 1516 urban and 1518 rural paediatric logs for analysis.

### How often do FLOs occur?

FLOs occurred in more than half of cases logged, with significantly more for rural compared to urban students (OR 1.35 95% CI 1.17, 1.56; *p* < 0.0001). For rural students, 666 (43.9%) of cases had no FLO, 618 (40.7%) had one FLO and 234 (15.4%) had more than one FLO; for urban students 778 (51.3%) had no FLO, 612 (40.4%) had one FLO and 125 (8.2%) had more than one FLO (*p* < 0.0001). Major FLOs occurred in over a third of cases logged, but with no significant difference between rural and urban logs (OR 1.10 95% CI 0.94, 1.28; *p* = 0.24).

### FLO categories for all logs

Medical FLOs were the most common, accounting for 64.0% (545/852) of all rural and 75.2% (555/738) of all urban FLOs (OR 1.71 95%CI 1.37, 2.12; *p* < 0.0001). Rural students had significantly more FLOs relating to professionalism issues (14.6% v 7.1%, OR 2.23 95% CI 1.75, 2.84; *p* < 0.001); insufficient clinical information (8.6% v 1.5%, OR 6.36 95% CI 4.02, 10.05, *p* < 0.001); relating to clinical reasoning (5.4% v 3.8%, OR 1.46 95% CI 1.03, 2.07; *p* = 0.03); and regarding student wellbeing (0.5% v none *p* = 0.016). Urban students had significantly more quality and safety FLOs than rural students (4.8% v 2.2%, OR 2.28 95% CI 1.50, 3.46; *p* < 0.001).

### What were the case complexities of rural compared to urban paediatric logs?

Students logged paediatric cases with a variety of overall clinical complexities across all healthcare settings. Most cases logged by urban students in a tertiary healthcare setting were of primary and secondary complexity. Although the distribution patterns of case complexity appeared essentially the same, urban students logged significantly more cases of tertiary complexity than rural students (OR 1.30 95% CI 1.12, 1.51; *p* = 0.0018), shown in Table 1.

**Table 1:** Case complexity across healthcare settings for urban and rural case logs

	Case Clinical Complexity						Total %	
	Primary		Secondary		Tertiary			
Location	Rural	Urban	Rural	Urban	Rural	Urban	Rural	Urban
Emergency %	164 35.8%	237 33.5%	202 44.1%	294 41.6%	92 20.1%	176 24.9%	458 30.2%	707 46.6%
Inpatient %	117 16.9%	14 2.3%	353 50.9%	285 47.0%	223 32.2%	307 50.7%	693 45.7%	606 40.0%
Outpatient %	22 6.8%	17 11.2%	131 40.7%	41 27.0%	169 52.4%	94 61.8%	322 21.1%	152 10.0%
Other^a^ %	13 28.9%	5 9.8%	18 40.0%	34 66.7%	14 31.1%	12 23.5%	45 3.0%	51 3.4%
Total	316	273	704	654	498	589	1518	1516
% of cases	20.8%	18.0%	46.4%	43.1%	32.8%	38.9%		

^a^other included school, home, overseas electives

Case complexity for cases logged in emergency departments was the same regardless of the rural or urban setting, with more than a third of primary complexity. However, the patterns of inpatient and outpatient logs were different between urban and rural students, when analysed specifically. More than half the cases rated as tertiary complexity cases logged by urban students were inpatients. In contrast, more than a third of these highly complex cases were seen as outpatients by rural students. More than half the rural inpatient logs were of secondary level complexity, with significantly more primary, and fewer tertiary complexity inpatients than urban cases logged (*p* < 0.0001). Urban students logged significantly fewer outpatients of secondary complexity compared to rural.

### Did more complex cases result in more major FLOs?

Table 2 demonstrates the pattern of major FLOs by case complexity. Major medical FLOs increased with increasing patient complexity, particularly for urban students where one third of tertiary complexity logs had a major medical FLO (*p* < 0.001), with one fifth for rural students (OR 1.95 95% CI 1.47, 2.59). None of the other major FLO categories increased with increasing complexity.

**Table 2:** Major feedback learning opportunities (FLOs) by complexity

	Case Clinical Complexity							
	Primary (%)		Secondary (%)		Tertiary (%)		Total	
Major FLO	Rural	Urban	Rural	Urban	Rural	Urban	Rural	Urban
Any Major FLO	97 (30.7%)	61 (22.3%)	220 (31.3%)	214 (32.7%)	178 (35.7%)	251 (42.6%)	495 (32.6%)	526 (34.7%)
Medical	30 (9.5%)	30 (11.0%)	115 (16.3%)	138 (21.1%)	97 (19.5%)	189 (32.1%)	242 (15.9%)	357 (23.5%)
Professionalism	49 (15.5%)	19 (7.0%)	69 (9.8%)	37 (5.7%)	63 (12.7%)	43 (7.3%)	181 (11.9%)	99 (6.5%)
Clinical reasoning	15 (4.7%)	9 (3.3%)	41 (5.8%)	22 (3.4%)	22 (4.4%)	17 (2.9%)	78 (5.1%)	48 (3.2%)
Insufficient	20 (6.3%)	2 (0.7%)	65 (9.2%)	11 (1.7%)	36 (7.2%)	7 (1.2%)	121 (8.0%)	20 (1.3%)
Social	2 (0.6%)	3 (1.1%)	6 (0.9%)	10 (1.5%)	2 (0.4%)	15 (2.5%)	10 (0.7%)	28 (1.8%)
Quality and Safety	9 (2.8%)	13 (4.8%)	14 (2.0%)	34 (5.2%)	6 (1.2%)	23 (3.9%)	29 (1.9%)	70 (4.6%)
Wellbeing	0 (0%)	0 (0%)	1 (0.1%)	0 (0%)	6 (1.2%)	0 (0%)	7 (0.5%)	0 (0%)
Total cases	316	273	704	654	498	589	1518	1516

More major FLOs occurred in rural primary complexity cases logged compared to urban (OR 1.54 95% CI 1.06, 2.23; *p* = 0.025). There was no difference for secondary complexity cases (OR 0.93 95% CI 0.74, 1.17; *p* = 0.60). Consequently, fewer tertiary complexity cases logged by rural students had some form of FLO (OR 0.75 95% CI 0.59, 0.96; *p* = 0.021).

Inter-rater agreement for patient complexity with a rural general paediatrician was 0.95 (95% CI 0.86, 1.00) and for major FLO was 0.94 (CI 0.82, 1.00). Similarly, intra-rater agreements were 0.87 (0.73, 1.00) and 0.88 (0.72, 1.00) respectively.

## Discussion

Case logs are a valuable resource for medical educators to enhance students’ learning experiences by providing meaningful feedback. Feedback learning opportunities (FLOs) occurred often in medical student logs of paediatric patients, particularly in cases with multiple medical problems. This study strengthens recommendations for regular review [11] and timely feedback on student logs [12].

FLOs occurred often, and were mostly medical, fitting with previous research that students under-report medical conditions [9, 13]. We acknowledge that cases with any FLO may have benefited from review with a medical educator, however the feedback categories suggested in this paper present a framework for discussions. They provided information about a students’ understanding and were specific, addressed inaccuracies, identified where students may have needed advice, reinterpretation, and/or alternatives provided to their learning, as recommended in the feedback literature [1].

Rural students had more FLOs overall, however there was no difference in major FLOs between rural and urban students. The increased FLOs identified for rural students were minor, which may be explained by increased insufficient and professionalism FLOs by rural students. This has been addressed by amending logging requirements for rural students following this study, and are unlikely to have future implications.

This study demonstrates that urban and rural clinical schools in WA provide medical students with a variety of patient complexities. Students logged mostly primary and secondary complexity cases, in keeping with the curriculum learning outcomes for urban [14] and rural medical graduates [15].

Emergency departments (EDs) were frequently used by rural and urban students to log paediatric cases, with the same proportions of case complexities. Students logged many primary complexity cases logged in EDs, consistent with 15.4% of paediatric presentations to Australian EDs allocated Category 5, or “low urgency” using the Australasian Triage Scale [16].

Rural outpatient clinics, including visiting specialist clinics by paediatricians and subspecialists [17], were a valuable resource for rural students to log secondary and tertiary complexity paediatric cases [18]. Urban students underutilised outpatients, particularly secondary complexity cases.

Varying referral and admission patterns for rural patients in Australia [19, 20], may explain the different patterns of inpatient and outpatient logs between urban and rural students. These are relevant to rural medical students’ learning as the higher proportion of rural primary complexity inpatients reflects the health needs of the rural community.

A substantial proportion of tertiary complexity cases logged had a major medical FLO, which may relate to higher acuity patients, as most logs were of tertiary paediatric hospital inpatients. Feedback on paediatric patients with multiple medical problems is therefore recommended.

### Limitations

The contrasting logging methods used by rural and urban students could explain some of the differences observed in this study. Although students using electronic logs recorded more patient problems in one study [21], the overall influence of written or electronic logs on the accuracy of clinical information recorded is unclear [22].

Logs reflect one aspect of students’ actual learning experiences, so FLOs may have been overestimated as logs document only what the student has recorded. It is also possible students had received feedback from medical educators, or counselling, about confronting clinical scenarios logged.

Students’ own style and degree of student supervision are variables which influence student learning [6]. Awareness of the students’ learning approach (deep or surface) may have been useful as a surface learning approach may underlie some of the insufficient FLOs.

Analysing logs for FLOs assumes all students were given equivalent instruction on how to log using best practice by medical educators in urban and rural sites. The increased professionalism FLOs by rural students constituted only a small proportion of FLOs, so is noteworthy but not of concern, and may be related to the requirement for rural students to log 10% of cases relating to personal and professional development.

This study was not designed to determine if students chose to strategically log less complex patients. However, the finding that students logged mostly primary and secondary cases fits with clinical practice, so student choice was unlikely to have highly influenced the complexity of cases logged.

Interpretation of the logs as entered by the student was subjective, however the same criteria were used for urban and rural. The potential for bias was minimised, as HW has experience in urban and rural schools, and clinical settings.

Ideally, review of the patients’ medical records in parallel with the students’ logs would have provided more comprehensive clinical information, perhaps increasing the quantity of medical FLOs, and strengthening the recommendations for feedback.

As logs were de-identified this was not possible. However, the large number of logged cases analysed in this study allowed a greater breadth of FLO type and grade to be compared.

### Practical implications

The data presented in this paper suggests most logs of paediatric patients by rural and urban medical students in Western Australia contain opportunities for medical educators to provide them with feedback that may enhance their learning. Next steps are to ensure medical educators can recognise FLO’s in medical student logs and know how to give effective feedback to students once identified.

We acknowledge the detail undertaken in this study by the principal investigator goes beyond the scope of most marking processes, and the coding convention would need further testing before widespread use. Nonetheless, we anticipate most FLOs would be easily identified by medical educators.

We recommend using the FLOs categories of medical, professionalism, insufficient, clinical reasoning, student wellbeing, quality and safety, and sociocultural as a practical framework for medical educators to help identify FLOs and aid discussions.

Medical FLOs discussions could guide students to consider all aspects of the case [9] such as co-existing diagnoses, developmental assessment or adolescent health issues. Professionalism FLOs could stimulate conversations encouraging reflection, suggest use of medical terminology, and debating ethical issues to aid personal and professional development [23]. Clinical reasoning FLO discussions could assist students with diagnostic skills [7]. Insufficient FLOs could stimulate conversations about ensuring relevant details are included. Sociocultural FLOs may result in discussions about cigarette smoking, the impact of living in a remote area, or relevant cultural issues. Quality and safety FLOs may emphasise the importance of patient safety in medical student education [24]. Wellbeing FLOs may identify students who should meet a medical educator for academic or personal support, if required [22].

This study examined logs from one clinical discipline and FLOs may occur with similar frequency in other disciplines and Schools. Further research using the methods described, after refining and testing the coding convention, could be undertaken to confirm this.

## Conclusions

Student logs of paediatric patients contain many feedback learning opportunities, so timely review and feedback is strongly recommended for their educational benefit to be realised. Student logs evidence clinical encounters but more importantly, students’ current thinking. Logs can provide teachers with opportunities to have meaningful conversations with students during feedback sessions. These data indicate that there is an educational requirement for medical educators to review cases logged to enhance the learning experience.

## Appendix

Feedback learning opportunities (FLO) scoring, and examples

TFOs were scored from 0 to 5 (0 – none, 1 – one minor, 2 multiple minor, 3 one major, 4 more than one major and 5 incorrect/dangerous information in the student log.

## Medical

“Could have reviewed tracheo-oesophageal fistula and potential complications.”

“Could have reviewed allergic rhinitis and ADHD.”

“Should have explored eczema.”

“Stridor not explored.”

## Professionalism

“Review of this log book an opportunity to improve written communication.”

“..concerning terminology that could be addressed by review of their logging.”

## Insufficient

“Should have more detail.”

“Minimal info in case.”

“Insufficient examination.”

## Clinical reasoning

“Incorrect diagnosis concern.”

“Needed to include pertussis in differential.”

## Wellbeing

“Student needs debrief post child sexual assault case.”

“Student needs debrief.” – post stillbirth.

## Quality and safety

“Unsafe to give trimeprazine to under 2 year old.”

## Socio-cultural

“Opportunity to reflect on issues for Aboriginal child from rural community.”

“Should have smoking history in child with multiple asthma presentations.”

“Social history required for recurrent ear disease.”

“Should have identified need to use an interpreter.”

## Abbreviations

AIHW: Australian Institute of Health and Welfare
FLOs: Feedback learning opportunities
MDCs: Major diagnostic categories
RCSWA: Rural Clinical School of Western Australia
UWA: University of Western Australia
